# Enterocutaneous Fistula and Abscess Diagnosed with Point-of-care Ultrasound

**DOI:** 10.5811/cpcem.2021.4.49918

**Published:** 2021-10-05

**Authors:** Sarah McCuskee, Kenton L. Anderson

**Affiliations:** * New York University School of Medicine, Department of Emergency Medicine, New York, New York; † Stanford University School of Medicine, Department of Emergency Medicine, Palo Alto, California

**Keywords:** ultrasound, fistula, hernia repair

## Abstract

**Case Presentation:**

A 64-year-old female with history of umbilical hernia repair with mesh 18 years prior, cystocele, and diabetes mellitus presented with 10 days of abdominal and flank pain. The patient was tachycardic, normotensive, afebrile, and had an erythematous, tender, protuberant abdominal wall mass. Point-of-care ultrasound (POCUS) revealed an irregular, heterogeneous extraperitoneal fluid collection with intraperitoneal communication; these findings were consistent with an abscess and infected mesh with evidence for intraperitoneal extension. The diagnosis of enterocutaneous fistula (ECF) with infected mesh and abdominal wall abscess was confirmed with computed tomography and the patient was admitted for antibiotics and source control.

**Discussion:**

A rare complication of hernia repair with mesh, ECF typically occurs later than more common complications including cellulitis, hernia recurrence, and bowel obstruction. In the emergency department, POCUS is commonly used to evaluate for abscess; in other settings, comprehensive ultrasound is used to evaluate for complications after hernia repair with mesh. However, to date there is no literature reporting POCUS diagnosis of ECF or mesh infection. This case suggests that distant surgery should not preclude consideration of mesh infection and ECF, and that POCUS may be useful in evaluating for these complications.

## CASE PRESENTATION

**Image 1:**
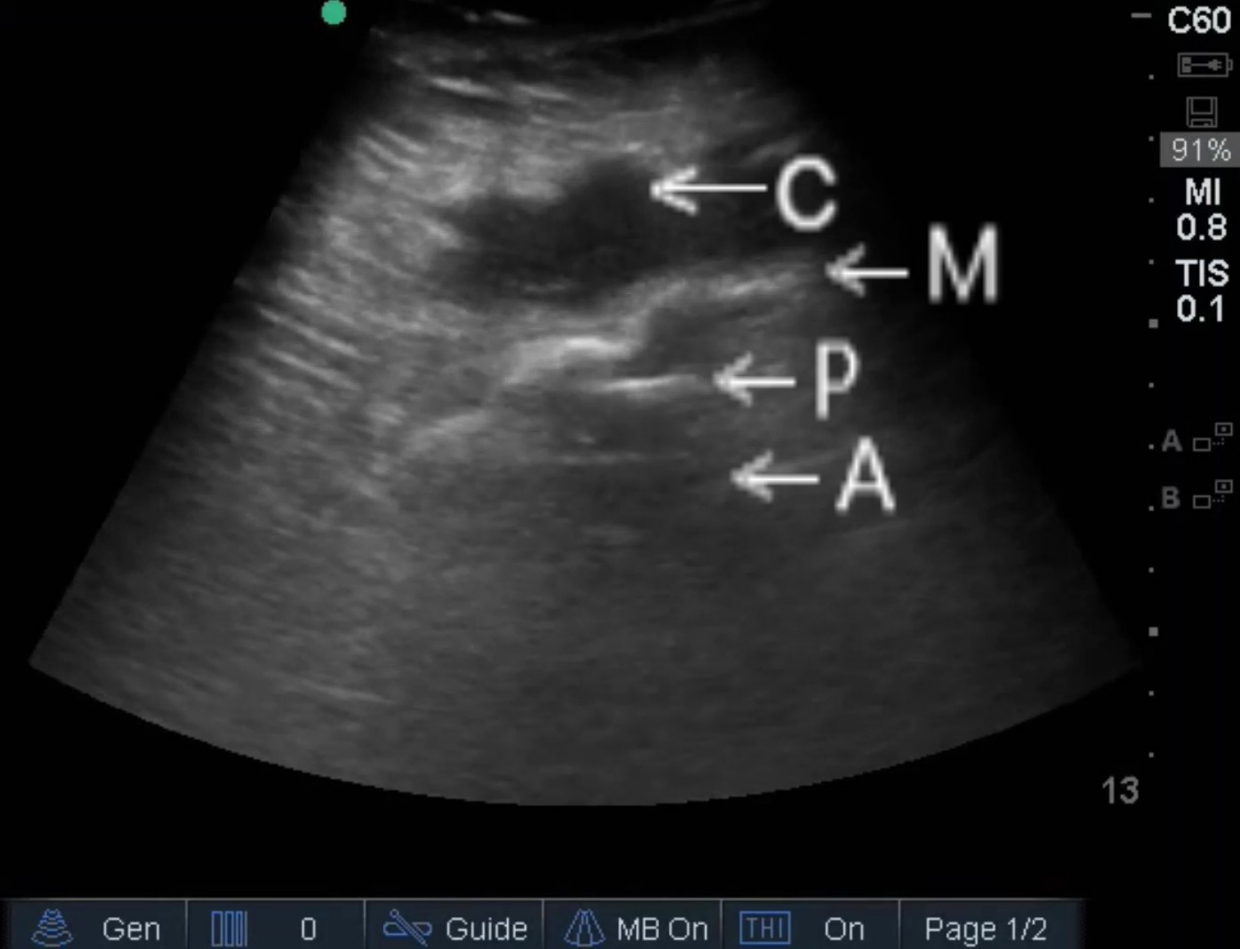
Point-of-care ultrasound of the abdominal wall abscess, demonstrating surgical mesh (M) and associated fluid collection (C) extending across the peritoneum (P) and communicating with intraperitoneal abscess (A).

A 64-year-old female with history of umbilical hernia repair with mesh 18 years prior, cystocele, and diabetes mellitus presented to the emergency department (ED) with 10 days of abdominal and flank pain. She also had alternating constipation and diarrhea, nausea, anorexia, and chills. She delayed her presentation due to cost. At presentation, the patient was tachycardic (heart rate 116 beats per minute), normotensive (blood pressure 106/58 millimeters of mercury), and afebrile. Physical examination revealed a five-centimeter erythematous, tender, abdominal wall mass. The initial differential diagnosis included incarcerated hernia, diverticulitis, and abscess. Point-of-care ultrasound (POCUS) revealed an irregular, heterogenous, extraperitoneal fluid collection with intraperitoneal communication, consistent with abdominal wall abscess extending beyond the surgical mesh into the peritoneum (Video, Image 1).

**Image 2:**
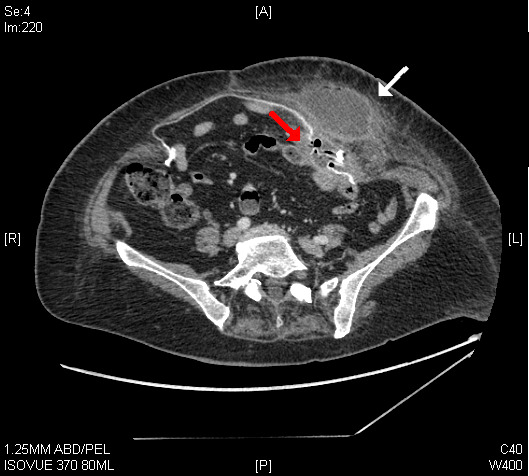
Computed tomography showing extraperitoneal (white arrow) and intraperitoneal (red arrow) abscesses associated with mesh and small intestine.

Computed tomography confirmed the enterocutaneous fistula (ECF) and abscess secondary to mesh migration and erosion into the small intestine (Image 2). The patient was admitted for intravenous antibiotics. Drain placement produced 90 milliliters of feculent pus.

## DISCUSSION

Erosion of surgical mesh into the intestinal wall and subsequent ECF formation is a rare complication of hernia repair with mesh. One series of 695 patients with mean follow-up of 4.9 years developed no ECFs,^1^ while 3.5% of a 200-patient series followed for a mean of 6.7 years developed ECFs at 3.3 years median postoperative time.^2^ Fistula formation occurred later than more common complications including cellulitis, hernia recurrence, and bowel obstruction. Treatment involves resecting the fistula, associated intestine, and mesh.^3^

Abscesses are frequently diagnosed with POCUS in the emergency department;^4^ however, to date no literature reports POCUS diagnosis of ECF or mesh infection. Comprehensive ultrasonography is used to diagnose mesh infections after hernia repair.^5^ Abscess due to mesh infection is sonographically similar to abscess from other sources; mesh and bowel function are particularly well visualized with sonography.^5^ This case suggests that distant surgery should not preclude consideration of mesh infection and ECF, and that POCUS is useful in evaluating for these complications.

**CPC-EM Capsule**

What do we already know about this clinical entity?

*Enterocutaneous fistula (ECF) is a rare complication of hernia repair with surgical mesh, which occurs later than other surgical complications.*

What is the major impact of the image(s)?

*This image demonstrates ECF occurring 18 years after hernia repair, diagnosed using point-of-care ultrasound in the emergency department.*

How might this improve emergency medicine practice?

*Distant surgery does not rule out mesh infection and ECF, and point-of-care ultrasound may be useful in making the diagnosis.*

## Supplementary Information

Video. Point-of-care ultrasound of the abdominal wall abscess, demonstrating a heterogenous fluid collection (C) associated with surgical mesh (M) extending across the peritoneum (P) and communicating with intraperitoneal abscess (A).

## References

[1] Brandi CD, Roche S, Bertone S (2017). No enterocutaneous fistula development in a cohort of 695 patients after incisional hernia repair using intraperitoneal uncoated polyproylene mesh. Hernia.

[2] Leber GE, Garb JL, Alexander AI (1998). Long-term complications associated with prosthetic repair of incisional hernias. Arch Surg.

[3] Costa D, Tomás A, Lacueva J (2004). Late enterocutaneous fistula as a complication after umbilical hernioplasty. Hernia.

[4] Tayal VS, Hasan N, Norton HJ (2006). The effect of soft-tissue ultrasound on the management of cellulitis in the emergency department. Acad Emerg Med.

[5] Crespi G, Giannetta E, Mariani F (2004). Imaging of early postoperative complications after polypropylene mesh repair of inguinal hernia. Radiol Med.

